# EHMTI-0254. One- year follow up study of clinical characteristics of headache and its profile in a case series of cerebral venous sinus thrombosis

**DOI:** 10.1186/1129-2377-15-S1-C17

**Published:** 2014-09-18

**Authors:** A Ghabeli Juibary, M Farzadfard, S Yazdani

**Affiliations:** 1 Department of Neurology, Ghaem Educational HospitalMashhad University of Medical Sciences (MUMS), Mashhad, Iran

## Introduction

Headache is the most frequent presenting symptom of cerebral venous thrombosis (CVT), most commonly associated with other manifestations.

## Aims

We investigated the pattern and location of headache in consecutive sixty patients with confirmed diagnosis of CVT to elucidate clinical characteristics of headache and its profile.

## Methods

Patients with diagnosis of CVT referring to Ghaem hospital neurology department a referral neurology center in north eastern Iran (Mashhad- Iran) were recruited consecutively during one year and followed for 12 months. Diagnosis of CVT was made by magnetic resonance imaging (MRI) combined with MR venography (MRV): both increased signal on MRI T1 and T2 weighted images and the absence of flow was required to confirm diagnosis.

## Results

Age range was 18–83 years (mean: 38.11 years). On presentation, study of location of headache showed 34(56.7%) had diffuse headache, 10(16.7%) frontal, 7(11.7%) hemi cranial, 5(8.3%) temporal and two (3.3%) patients had temporal and occipital headaches. Nine (15%) patients presented with thunder club headache. Only one patient had persistent headache after one year follow up. 51(85%) patients had papilleadema. Duration of headache in 36(60%) patients was between three to seven days.

## Conclusions

There is no special, consistent, identifiable pattern of headache in CVT patients.

No conflict of interest.

